# Comparative Analysis of Culture and Sputum Smear Conversion Timelines and Their Associated Factors in Smokers Versus Non-smokers With Drug-Resistant Tuberculosis

**DOI:** 10.7759/cureus.80704

**Published:** 2025-03-17

**Authors:** Sajjad Ali, Nabi Rahman Rahman, Ashraf, Rumman, Akmal Naveed, Abdul Ghafoor, Murad Ali, Ubaid Ullah

**Affiliations:** 1 Pulmonology, Medical Teaching Institution (MTI) Mardan Medical Complex, Mardan, PAK; 2 Research and Development, Pro-Gene Diagnostics and Research Laboratory, Mardan, PAK; 3 Pulmonary, Medical Teaching Institution (MTI) Mardan Medical Complex, Mardan, PAK; 4 Pharmacovigilance/Active Drug Safety Monitoring and Management, Association for Community Development, Peshawar, PAK; 5 Association for Community Development, Peshawar, PAK; 6 Multidrug-Resistant Tuberculosis (MDR-TB) Department, Koninklijke Nederlandse Centrale Vereniging (KNCV) Pakistan, Lahore, PAK; 7 Medicine, Bacha Khan Medical College, Mardan, PAK; 8 Tuberculosis, Association For Community Development, Peshawar, PAK; 9 Department of Pulmonology, Mardan Medical Complex, Mardan, PAK

**Keywords:** culture conversion timeline, drug-resistant tuberculosis (dr-tb), smoker, smoking and treatment outcomes, sputum smear conversion

## Abstract

Background

Drug-resistant tuberculosis (DR-TB) is a global health challenge, with smoking potentially affecting treatment outcomes. Smoking compromises immune function and may interfere with the pharmacokinetics of anti-TB drugs. Delayed sputum smear and culture conversion are key indicators of prolonged treatment and infectiousness. This study explores the impact of smoking on these conversion timelines in DR-TB patients.

Objective

To identify and assess the overall treatment outcomes and the factors associated with delayed culture and sputum smear conversion in smokers compared to non-smokers among patients with drug-resistant tuberculosis.

Methods

This prospective cohort study was conducted at the Programmatic Management of Drug-Resistant TB (PMDT) unit at Mardan Medical Complex in Khyber-Pakhtunkhwa, Pakistan, from June 2020 to December 2024. All patients diagnosed with drug-resistant tuberculosis (DR-TB) were categorized into two groups based on their smoking status: smokers and non-smokers. Patient demographic and clinical information was collected through structured interviews and standardized questionnaires. The time to sputum smear and culture conversion (SCC) was longitudinally measured from the start of treatment until the patient achieved two consecutive negative smears and three consecutive negative cultures, respectively. Cox proportional hazards analysis was employed to evaluate the relationship between smoking status and time to SCC, adjusting for potential confounding factors. Kaplan-Meier survival curves were used to compare the time to SCC between the two groups, with statistical significance set at p < 0.05. Analyses were performed using SPSS software (version 29.0).

Results

Out of 281 DR-TB patients, 138 were smokers (49.12%) and 143 were non-smokers (50.88%). Non-smokers achieved faster sputum and culture conversion, with survival proportions dropping to 0.000 by 90 and 70 days, respectively. In contrast, smokers showed slower declines, with sputum conversion at 0.137 and culture conversion at 0.035 by 120 days. The mean sputum conversion time was 59 days for non-smokers and 104 days for smokers, while culture conversion took 43 days for non-smokers and 98 days for smokers. Multivariate analysis identified significant determinants for both groups: older age (≥36 years), lower BMI (<16 kg/m²), and higher sputum smear grades. Non-smokers were adversely affected by gastrointestinal upset and nephrotoxicity, while smokers were more negatively impacted by higher cigarette consumption, diabetes, and lung lesions. Long-term treatment regimens and resistance to antibiotics like levofloxacin and moxifloxacin reduced conversion rates in both groups.

Conclusion

Smoking not only impairs immune function but also influences the pharmacokinetics of anti-TB drugs, potentially leading to more prolonged and complicated treatment courses.

## Introduction

Tuberculosis (TB) continues to be a significant global health concern, with around 10 million new cases and 1.4 million deaths reported in 2019 alone [1]. The situation is further complicated by the emergence of drug-resistant TB, particularly multidrug-resistant TB (MDR-TB), which presents greater challenges and higher treatment costs [2]. Additionally, comorbid factors such as smoking have been recognized as potential influencers of TB treatment outcomes [3].

Drug-resistant TB presents significant public health challenges due to its complex treatment regimens, longer treatment durations, and lower success rates compared to drug-susceptible TB [4]. A crucial milestone in TB management is the timely conversion of culture and sputum smear from positive to negative, which indicates treatment effectiveness and reduces transmission risk [5]. Delays in these conversion timelines suggest suboptimal treatment responses and are linked to poorer outcomes.

Studies have shown that smoking negatively impacts TB treatment outcomes. Smokers with TB are more likely to experience treatment failure, relapse, and mortality compared to non-smokers [6]. The immunosuppressive effects of smoking impair the body’s response to TB infection and treatment, leading to prolonged disease and infectiousness [7]. Additionally, smoking is associated with lower adherence to TB treatment regimens, complicating disease management.

For drug-resistant TB, the challenges are even greater. Smokers with MDR-TB face worse treatment outcomes, including longer times to culture conversion and higher rates of treatment default and mortality compared to non-smokers [6]. However, these studies often do not thoroughly investigate the specific factors contributing to these differences, indicating a need for more focused research.

The conversion of sputum smear and culture to negative is a key indicator of TB treatment success. Early conversion is linked to a lower risk of treatment failure and relapse, as well as reduced disease transmission [8]. Delayed conversion suggests ongoing bacterial activity and may necessitate treatment adjustments [9]. Understanding the factors that influence these timelines in smokers versus non-smokers can provide critical insights for optimizing treatment strategies.

Despite the known adverse impacts of smoking on health and TB outcomes, research specifically on how smoking affects drug-resistant TB treatment outcomes is limited. Identifying the factors associated with delayed culture and sputum smear conversion in smokers compared to non-smokers could provide valuable insights for clinical practice and public health strategies. It is essential to pinpoint these factors to improve treatment protocols and outcomes for this vulnerable population.

This study aims to identify and assess the factors associated with delayed culture and sputum smear conversion in smokers versus non-smokers among patients with drug-resistant tuberculosis (DR-TB). By elucidating these factors, the study seeks to provide a foundation for targeted interventions that can enhance treatment outcomes for smokers with drug-resistant TB.

Understanding the factors associated with delayed culture and sputum smear conversion in smokers versus non-smokers can help explain the differential treatment outcomes observed in these groups. These insights are crucial for developing targeted interventions to improve treatment efficacy and patient prognosis. This study aims to fill the knowledge gap regarding the interaction between smoking and drug-resistant TB treatment, potentially informing more personalized treatment approaches and public health policies.

## Materials and methods

Study design and setting

A prospective cohort study was conducted at the Programmatic Management of Drug-Resistant TB (PMDT) unit at Mardan Medical Complex in Khyber-Pakhtunkhwa, Pakistan, from June 2020 to December 2024. This longitudinal study involved using a convenience sampling method to categorize patients with DR-TB into two groups based on their smoking status smokers and non-smokers to evaluate the impact of smoking on treatment outcomes over time.

Inclusion and exclusion criteria

The inclusion criteria for the study included patients with positive sputum smear and culture reports at the start of the study, a medically confirmed diagnosis of DR-TB, non-smokers, and both current and former smokers. Exclusion criteria consisted of patients with incomplete medical records, co-infections such as HIV or COVID-19, other major comorbidities like cancer or autoimmune diseases, unstable psychiatric conditions, and those who had undergone surgery within the previous three months.

Data collection

Demographic and clinical information was gathered through structured interviews and standardized questionnaires. Sputum samples for smear and culture tests were collected from each patient.

Definitions and conversion criteria

Sputum smear conversion was defined as two consecutive negative smears, while culture conversion was defined as three consecutive negative cultures taken at intervals of at least 30 days. The time to sputum smear and culture conversion (SCC) was recorded in days.

Treatment regimens

Patients were assigned to either a long-term treatment regimen (18-24 months) or a short-term treatment regimen (9-12 months) based on the latest World Health Organization (WHO) guidelines. [9] A total of seven regimens were used (Table 1).

**Table 1:** Regimens used

Regimen	Drugs Included
Regimen A	Moxifloxacin, Ethionamide, Cycloserine, Linezolid, Delamanid, Para-amino Salicylic Acid, Pyrazinamide
Regimen B	Amikacin, Moxifloxacin, Ethionamide, Clofazimine, Pyrazinamide, High-Dose Isoniazid, Ethambutol
	or Moxifloxacin, Clofazimine, Pyrazinamide, Ethambutol
Regimen C	Bedaquiline, Levofloxacin, Linezolid, Cycloserine, Clofazimine, Pyrazinamide
	or Levofloxacin, Cycloserine, Clofazimine, Pyrazinamide
Regimen D	Bedaquiline, Moxifloxacin/Levofloxacin, Ethionamide, Clofazimine, Pyrazinamide, High-Dose Isoniazid, Ethambutol
	or Moxifloxacin/Levofloxacin, Clofazimine, Pyrazinamide, Ethambutol
Regimen E	Pyrazinamide, Amikacin, Moxifloxacin/Levofloxacin, Cycloserine, Ethionamide, Linezolid
	or Pyrazinamide, Moxifloxacin/Levofloxacin, Cycloserine, Ethionamide, Linezolid
Regimen F	Pyrazinamide, Amikacin/Capreomycin, Moxifloxacin, Cycloserine, Ethionamide, Linezolid
	or Moxifloxacin, Cycloserine, Ethionamide, Linezolid
Regimen G	Bedaquiline, Amikacin/Delamanid, Linezolid, Clofazimine, Cycloserine, Pyrazinamide
	or Linezolid, Clofazimine, Cycloserine, Pyrazinamide

Statistical analysis

Continuous variables were summarized as means and standard deviations, while categorical variables were reported as frequencies and percentages. Differences between baseline characteristics of the two groups were analyzed using the chi-squared test for categorical variables and the t-test or Mann-Whitney U test for continuous variables, depending on the data distribution. Hazard ratios (HR) with 95% confidence intervals (CI) were calculated using Cox proportional hazards models to assess the impact of smoking status on time to sputum SCC, adjusting for potential confounders. Multivariable analysis was employed to identify independent predictors of time to sputum SCC in both smoker and non-smoker patients. Kaplan-Meier survival analysis was used to evaluate the impact of smoking status on the time to sputum SCC. Survival curves were plotted for both groups to compare the time to culture conversion, and mean days were calculated for each group. A survival table was also generated to summarize the survival times at different intervals. A p-value of less than 0.05 was considered statistically significant. All statistical analyses were performed using SPSS software (version 29.0).

Ethical considerations

The study was approved by the Institutional Review Boards of Mardan Medical Complex and Bacha Khan Medical College in Pakistan (Approval number: 336/BKMC). Participants provided fully informed consent and were free to join or withdraw from the study at any time.

## Results

The baseline characteristics of 281 DR-TB patients, comprising 138 smokers (49.12%) and 143 non-smokers (50.88%), reveal notable differences. The mean age of smokers was higher at 39.58 years (±14.42) versus 35.05 years (±18.42) for non-smokers (p = 0.020). Smokers had a lower mean BMI of 15.82 (±3.92) compared to 17.07 (±4.32) for non-smokers (p = 0.046). Marital status showed that 80.43% of smokers were married compared to 50.35% of non-smokers (p = 0.003). Clinical presentations were more severe in smokers, with a higher prevalence of lung lesions (94.93% vs. 72.73%, p = 0.001) and lung cavities (57.97% vs. 22.38%, p = 0.010), and higher sputum smear grades >1 (86.23% vs. 58.74%, p = 0.020). Comorbidities were also more prevalent among smokers, particularly diabetes mellitus (26.09% vs. 18.18%, p = 0.050), and heart diseases (6.53% vs. 1.29%, p = 0.045) (Table 2).

**Table 2:** Baseline characteristics of smokers vs non-smokers among drug-resistant tuberculosis patients Statistical analysis was performed using the chi-square (χ²) test for categorical variables and the t-test for continuous variables. P-value <0.05 is statically significant. P-values with *** represent no statistics were computed because the characteristic is either present in 100% or 0% of the population. “Smoking to Other Household Members” refers to whether the patient smokes in the presence of other individuals living in the same household. It indicates whether the patient’s smoking habits potentially expose other household members to secondhand smoke.

Characteristics	All Patients	Smokers	Non-smokers	p-value	Test Statistic
Total no of patients	281 (100)	138 (49.12)	143 (50.88)	***	
Gender					
Male	183 (65.12)	138 (100)	45 (31.47)	0.00	χ² = 89.79
Female	98 (34.88)	00 (0.00)	98 (68.53)		
Age	36.14±17.58	39.58±14.42	35.05±18.42	0.020	t = 2.029
5-14	04 (1.42)	00 (0.00)	04 (2.80)		
15-24	81 (28.83)	28 (20.29)	53 (37.06)		
25-34	55 (19.57)	35 (25.36)	20 (13.99)		
35-44	31 (11.03)	16 (11.59)	15 (10.49)		
45-54	55 (19.57)	36 (26.09)	19 (13.29)		
55-64	26 (9.25)	11 (7.97)	15 (10.49)		
>65	29(10.32)	12 (8.70)	17 (11.89)		
Body Mass Index	16.44±3.78	15.82±3.92	17.07±4.32	0.046	t = -2.019
<18.5	183 (65.12)	92 (66.67)	91 (63.64)		
18.5-24.9	52 (18.51)	33 (23.91)	19 (13.64)		
25.0-29.9	07 (2.49)	03 (2.17)	04 (2.80)		
30.0-35.9	00 (0.00)	00 (0.00)	00 (0.00)		
Living condition					
Rural	254 (90.39)	123 (89.13)	131 (91.61)	0.002	χ² = 8.11
Urban	27 (9.61)	15 (10.87)	12 (8.39)		
Marital status					
Married	183 (65.12)	111 (80.43)	72 (5035)	0.003	χ² = 8.85
Unmarried	98 (34.88)	27 (19.57)	71 (49.65)		
Smoker characteristics					
Smoking to Other Household Members	91 (32.38)	91 (65.94)	00 (0.00)		t = 1.98
Number of cigarette smoking per day	12.52±4.99	12.52±4.99	0.00±0.00	0.050	
Chest X-Ray Characteristics					
Lung lesions	235 (83.63)	131 (94.93)	104 (72.73)	0.001	χ² = 19.45
Lung cavities	112 (39.86)	80 (57.97)	32 (22.38)	0.010	χ² = 6.69
Comorbidities					
Diabetes mellitus	62 (22.06)	36 (26.09)	26 (18.18)	0.050	χ² = 3.84
Hypertension	08 (2.85)	02 (1.45)	06 (4.20)	0.305	χ² = 2.37
Liver diseases	09 (3.20)	07 (5.07)	02 (1.40)	0.159	χ² = 1.88
Heart diseases	11 (3.91)	09 (6.52)	02 (1.39)	0.045	χ² = 4.02
Renal diseases	03 (1.07)	02 (1.45)	01 (0.70)	0.974	χ² = 0.00
Sputum smear grads					
>1	203 (72.24)	119 (86.23)	84 (58.74)	0.020	χ² = 4.99
≤1	78 (27.76)	35 (25.36)	43 (30.07)		

The study observed 145 (51.60%) MDR-TB patients and 136 (48.40%) Xpert MTB/Rif Res patients. Smokers had a higher history of Category I tuberculosis treatment (63.77% vs. 48.95%, p = 0.005). Treatment regimens varied, but no significant differences were observed between smokers and non-smokers in the use of long-term (44.20% vs. 42.66%) and short-term regimens (55.80% vs. 57.34%). In terms of treatment outcomes, smokers had a lower success rate (77.53%) compared to non-smokers (92.30%, p = 0.002). The cure rate was also lower for smokers (37.68% vs. 45.45%, p = 0.001). Complete treatment rates were higher among non-smokers (46.85% vs. 39.85%, p = 0.003). Failures were slightly higher in smokers (1.45% vs. 0.70%, p = 0.011). The mortality rate was significantly higher in smokers (14.49% vs. 3.50%, p = 0.001). Loss of follow-up was higher in smokers (6.52% vs. 3.49%, p = 0.001) (Table 3).

**Table 3:** Treatment history, regimens, and outcomes of smokers vs. non-smokers with drug-resistant tuberculosis The chi-square (χ²) test was used for categorical variables. P-value <0.05 is statically significant. P-values with *** represent no statistics were computed because the characteristic is either present in 100% or 0% of the population. Xpert MTB/Rif Res: Refers to the Xpert MTB/Rif Resistance test, a molecular diagnostic test used to detect Mycobacterium tuberculosis and its resistance to Rifampicin; CAT-I: Refers to Category I of TB treatment, typically used for new, drug-sensitive TB patients with a standard 6-month regimen; CAT-II: Refers to Category II of TB treatment, used for patients who have been previously treated for TB but experienced relapse, failure, or default, involving a more intensive treatment regimen.

Characteristics	All Patients	Smokers	Non-smokers	P-value	Test Statistic
Total no of patients	281 (100)	138 (49.12)	143 (50.88)	***	
Type of drug resistance tuberculosis now					
MDR	145 (51.60)	76 (55.07)	69 (48.28)	0.466	χ² = 0.53
Xpert MTB/Rif Res	136 (48.40)	69 (50.00)	65 (45.45)	0.623	χ² = 0.24
Previous Tuberculosis FLD Treatment History And Episode Type					
CAT-I	158 (56.23)	88 (63.77)	70 (48.95)	0.005	χ² = 7.76
CAT-II	60 (21.35)	26 (18.84)	34 (23.78)	0.285	χ² = 1.15
No history of ATT	63 (22.42)	28 (20.29)	39 (22.38)	0.884	χ² = 0.02
Treatment Regimen Now					
Long-term treatment regimen	122 (43.42)	61 (44.20)	61 (42.66)	0.682	χ² = 0.17
Regimen A	02 (0.71)	00 (0.00)	02 (1.40)	1.000	χ² = 0.00
Regimen C	49 (17.43)	30 (21.73)	19 (13.28)	0.047	χ² = 4.04
Regimen E	43 (15.30)	14 (10.14)	29 (20.28)	0.082	χ² = 3.04
Regimen F	05 (1.78)	03 (2.17)	02 (1.40)	0.681	χ² = 0.17
Regimen G	21 (7.47)	13 (9.42)	08 (5.59)	0.102	χ² = 2.56
Short-term treatment regimen	159 (56.59)	77 (55.80)	82 (57.34)	0.59	χ² = 0.29
Regimen B	53 (18.86)	27 (19.56)	26 (18.18)	0.414	χ² = 0.67
Regimen D	106 (37.72)	52 (37.68)	54 (37.76)	0.632	χ² = 0.01
Treatment Outcome					
Success rate cured + complete	239 (85.05)	107 (77.53)	132 (92.30)	0.002	χ² = 9.57
Cured	117 (41.63)	52 (37.68)	65 (45.45)	0.001	χ² = 9.92
Complete	122 (43.32)	55 (39.85)	67 (46.85)	0.003	χ² = 8.93
Failed	03 (1.07)	02 (1.45)	01 (0.70)	0.011	χ² = 6.67
Died	25 (8.90)	20 (14.49)	05 (3.50)	0.001	χ² = 13.12
Loss of follow-up	14 (4.98)	09 (6.52)	05 (3.49)	0.001	χ² = 8.91

The most significant finding in the drug susceptibility testing (DST) results is the notably higher resistance to isoniazid and moxifloxacin among smokers compared to non-smokers with DR-TB. Specifically, 70.29% (97 out of 138) of smokers were resistant to isoniazid, compared to 32.17% (46 out of 143) of non-smokers (p = 0.000). Additionally, resistance to moxifloxacin was observed in 10.14% (14 out of 138) of smokers, versus 3.50% (5 out of 143) of non-smokers (p = 0.006) (Table 4).

**Table 4:** Drug susceptibility testing (DST) results of smokers vs. non-smokers with drug-resistant tuberculosis The chi-square (χ²) test was used for categorical variables to assess the differences between the two groups. P-value <0.05 is statically significant. P-values with *** represent no statistics were computed because the characteristic is either present in 100% or 0% of the population.

Characteristics	DST	All Patients	Smokers	Non-smokers	P-value	Test Statistic
Total no of patients		281 (100)	138 (49.12)	143 (50.88)	***	
Rifampicin	Sensitive	00 (0.00)	00 (0.00)	00 (0.00)	***	
	Resistant	281 (100)	138 (100)	143 (100)	0.749	χ² = 0.11
Isoniazid	Sensitive	87 (30.96)	41 (29.71)	97 (67.83)	0.000	χ² = 48.23
	Resistant	194 (69.04)	97 (70.29)	46 (32.17)	0.000	χ² = 48.23
Ethambutol	Sensitive	255 (90.75)	128 (92.75)	127 (88.81)	1.000	χ² = 0.00
	Resistant	26 (9.25)	10 (7.25)	16 (11.19)	0.325	χ² = 1.00
Pyrazinamide	Sensitive	252 (89.68)	124 (89.86)	128 (89.51)	0.801	χ² = 0.06
	Resistant	29 (10.32)	14 (10.14)	15 (10.49)	1.000	χ² = 0.00
Streptomycin	Sensitive	244 (86.83)	117 (84.78)	127 (88.81)	0.442	χ² = 0.59
	Resistant	37 (13.17)	21 (15.22)	16 (11.19)	0.497	χ² = 0.48
Amikacin	Sensitive	277 (98.58)	136 (98.55)	141 (98.60)	0.741	χ² = 0.04
	Resistant	04 (1.42)	02 (1.45)	02 (1.40)	1.000	χ² = 0.00
Levofloxacin	Sensitive	222 (79.00)	110 (79.71)	112 (78.32)	0.931	χ² = 0.02
	Resistant	59 (21.00)	28 (20.29)	31 (21.68)	0.786	χ² = 0.07
Moxifloxacin	Sensitive	262 (93.24)	124 (89.86)	138 (96.50)	0.271	χ² = 1.22
	Resistant	19 (6.76)	14 (10.14)	05 (3.50)	0.006	χ² = 7.43
Ethionamide	Sensitive	278 (98.93)	135 (97.83)	143 (100)	0.557	χ² = 0.35
	Resistant	03 (1.07)	03 (2.17)	00 (0.00)	1.000	χ² = 2.33
Clofazimine	Sensitive	279 (99.29)	138 (100)	141( 98.60)	0.871	χ² = 0.02
	Resistant	02 (0.71)	00 (0.00)	02 (1.40)	1.000	χ² = 2.33

The adverse effects observed in 281 DR-TB patients reveal significant differences between smokers and non-smokers. Nausea and vomiting were less prevalent among smokers (28.26%, 39 patients) compared to non-smokers (39.86%, 57 patients, p = 0.004). Urine discoloration was more common in smokers (92.03%, 127 patients) than non-smokers (78.32%, 112 patients, p = 0.002). Retinopathy occurred more frequently in smokers (28.99%, 40 patients) compared to non-smokers (16.78%, 24 patients, p = 0.046) (Table 5).

**Table 5:** Adverse effects in smokers vs. non-smokers with drug-resistant tuberculosis The chi-square (χ²) test was used for categorical variables to determine statistical differences. P-value <0.05 is statically significant. P-values with *** represent no statistics were computed because the characteristic is either present in 100% or 0% of the population.

Characteristics	Symptoms Presentation	All Patients	Smokers	Non-smokers	P-value	Test Statistic
Total no of patients		281 (100)	138 (49.12)	143 (50.88)	***	
Nausea and vomiting	Yes	96 (34.16)	39 (28.26)	57 (39.86)	0.004	χ² = 8.25
	No	185 (65.84)	99 (71.74)	86 (60.14)	0.281	χ² = 1.12
Peripheral Neuropathy	Yes	78 (27.76)	32 (23.19)	46 (32.17)	0.113	χ² = 2.45
	No	203 (72.24)	106 (76.81)	97 (67.83)	0.482	χ² = 0.50
Hepatotoxicity	Yes	102 (36.30)	44 (31.88)	58 (40.56)	0.155	χ² = 2.02
	No	179 (63.70)	94 (68.12)	85 (59.44)	0.469	χ² = 0.51
Drug-induced lupus	Yes	02 (0.71)	01 (0.72)	01 (0.70)	1.000	χ² = 0.00
	No	279 (99.29)	137 (99.28)	142 (99.30)	0.736	χ² = 0.00
Urine discoloration	Yes	239 (85.05)	127 (92.03)	112 (78.32)	0.232	χ² = 1.44
	No	42 (14.95)	11 (7.97)	31 (21.68)	0.002	χ² = 9.68
Gastrointestinal upset	Yes	120 (42.70)	52 (37.68)	68 (47.55)	0.123	χ² = 2.33
	No	161 (57.30)	86 (62.32)	75 (52.45)	0.351	χ² = 0.84
Hyperuricemia	Yes	36 (12.81)	14 (10.14)	22 (15.38)	0.228	χ² = 1.45
	No	245 (87.19)	124 (89.86)	121 (84.62)	0.865	χ² = 0.04
Arthralgia	Yes	85 (30.25)	35 (25.36)	50 (34.97)	0.099	χ² = 2.67
	No	196 (69.75)	103 (74.64)	93 (65.03)	0.426	χ² = 1.68
Optic neuritis	Yes	02 (0.71)	01 (0.72)	01 (0.70)	1.00	χ² = 0.00
	No	279 (99.29)	137 (99.28)	142 (99.30)	0.736	χ² = 0.00
Skin rash	Yes	51 (18.15)	24 (17.39)	27 (18.88)	0.769	χ² = 0.07
	No	230 (81.85)	114 (82.61)	116 (81.12)	0.932	χ² = 0.01
Central nervous system effects	Yes	00 (0.00)	00 (0.00)	00 (0.00)	***	
	No	281 (100)	138 (100)	143 (100)	***	
Nephrotoxicity	Yes	19 (6.76)	07 (5.07)	12 (8.39)	0.351	χ² = 1.04
	No	262 (93.24)	131 (94.93)	131 (91.61)	1.000	χ² = 0.00
Ototoxicity	Yes	33 (11.74)	13 (9.42)	20 (13.99)	0.282	χ² = 1.16
	No	248 (88.26)	125 (90.58)	123 (86.01)	0.932	χ² = 0.01
Anxiety	Yes	44 (15.66)	18 (13.04)	26 (18.18)	0.272	χ² = 1.23
	No	237 (84.34)	120 (86.96)	117 (81.82)	0.864	χ² = 0.02
Depression	Yes	102 (36.30)	57 (41.30)	45 (31.47)	0.229	χ² = 1.44
	No	179 (63.70)	81 (58.70)	98 (68.53)	0.147	χ² = 2.00
Retinopathy	Yes	64 (22.78)	40 (28.99)	24 (16.78)	0.046	χ² = 7.44
	No	217 (77.22)	98 (71.01)	119 (83.22)	0.043	χ² = 7.44

A multivariate analysis of smokers and non-smokers with DR-TB reveals distinct differences in the factors influencing sputum and culture conversion between the two groups. For both smokers and non-smokers, older age (≥36 years; HR 1.02, p = 0.038 for smokers, HR 1.00, p = 0.047 for non-smokers), lower BMI <16 kg/m²; HR 1.001, p = 0.048 for smokers, HR 0.53, p = 0.049 for non-smokers), and higher sputum smear grades (>1; HR 0.73, p = 0.029 for smokers, HR 1.02, p = 0.041 for non-smokers) are associated with reduced conversion rates. Additionally, non-smokers experience significant negative impacts from gastrointestinal upset (HR 0.70, p = 0.049) and nephrotoxicity (HR 0.45, p = 0.010). In smokers, higher cigarette consumption (≥12 per day; HR 1.51, p = 0.033), diabetes (HR 0.68, p = 0.039), and lung lesions (HR 1.43, p = 0.045) exacerbate the negative outcomes. Both groups show decreased conversion rates with extended treatment regimens (HR 1.47, p = 0.046 for smokers, HR 0.89, p = 0.018 for non-smokers) and resistance to critical antibiotics such as levofloxacin (HR 1.74, p = 0.018 for smokers, HR 0.62, p = 0.031 for non-smokers) and moxifloxacin (HR 1.77, p = 0.040 for smokers, HR 0.29, p = 0.016 for non-smokers) (Tables 6-9).

**Table 6:** Multivariate analysis of factors influencing sputum conversion in smokers with drug-resistant tuberculosis P-value <0.05 is statically significant. STR: short-term regimen; LTR: long-term regimen; Xpert MTB/Rif Res: Refers to the Xpert MTB/Rif Resistance test, a molecular diagnostic test used to detect *Mycobacterium tuberculosis* and its resistance to Rifampicin; CAT-I: Refers to Category I of TB treatment, typically used for new, drug-sensitive TB patients with a standard 6-month regimen; CAT II: Refers to Category II of TB treatment, used for patients who have been previously treated for TB but experienced relapse, failure, or default, involving a more intensive treatment regimen.

Characteristic	Categories	Sputum Conversion		HR	95% CI	P-value
		Yes	No			
All patients	138	107 (77.53)	31 (22.47)			
Age	< 36	49 (45.79)	14 (45.16)	REF		
	≥ 36	58 (54.21)	17 (54.84)	1.02	0.99-1.12	0.038
Body mass index	≥ 16 kg/m²	51 (47.66)	07 (22.58)	REF		
	< 16 kg/m²	56 (52.34)	24 (77.42)	1.001	0.94-1.05	0.048
Living condition	Rural	94 (87.85)	17 (54.84)	REF		
	Urban	13 (12.15)	14 (45.16)	0.51	0.32-0.89	0.041
Number of cigarette smoking per day	< 12	54 (50.47)	18 (58.06)	REF		
	≥ 12	53 (49.53)	13 (41.94)	1.51	1.19-1.89	0.033
Lung lesions	No	20 (18.69)	03 (9.68)	REF		
	Yes	87 (81.31)	28 (90.32)	1.43	0.88-2.34	0.045
Lung cavities	No	43 (40.19)	15 (48.39)	REF		
	Yes	64 (59.81)	16 (51.61)	1.03	0.68-1.19	0.042
Diabetes mellitus	No	77 (71.96)	25 (80.65)	REF		
	Yes	30 (28.04)	06 (19.35)	0.68	0.45-0.91	0.039
Sputum smear grades	≤ 1	49 (45.79)	17 (54.84)	REF		
	>1	58 (54.21)	14 (45.16)	0.73	0.48-1.10	0.029
Drug resistance tuberculosis	Xpert MTB/Rif Res	48 (44.86)	20 (64.52)	REF		
	MDR	59 (55.14)	11 (35.48)	1.87	1.48-2.34	0.000
Treatment history	No history of ATT	25 (23.36)	03 (9.68)	REF		
	CAT-I	70 (65.42)	18 (58.06)	0.52	0.38-0.89	0.026
	CAT-II	16 (14.95)	10 (32.26)			
Treatment regimen now	STR	56 (52.34)	20 (64.52)	REF		
	LTR	51 (47.66)	11 (35.48)	1.47	1.00-2.16	0.046
Antibiotics						
Levofloxacin	Sensitive	83 (77.57)	26 (83.87)	REF		
	Resistant	24 (22.43)	05 (16.13)	1.74	1.09-2.76	0.018
Adverse Effects						
Nausea and vomiting	No	78 (72.90)	22 (70.97)	REF		
	Yes	29 (27.10)	09 (29.03)	0.62	0.40-0.96	0.034
Peripheral neuropathy	No	82 (76.64)	25 (80.65)	REF		
	Yes	25 (23.36)	06 (19.35)	0.50	0.31-0.80	0.004
Hepatotoxicity	No	72 (67.29)	23 (74.19)	REF		
	Yes	35 (32.71)	08 (25.81)	0.52	0.34-0.79	0.003
Arthralgia	No	81 (75.70)	23 (74.19)	REF		
	Yes	26 (24.30)	08 (25.81)	0.61	0.39-0.97	0.037

**Table 7:** Multivariate analysis of factors influencing culture conversion in smokers with drug-resistant tuberculosis P-value <0.05 is statically significant. STR: short-term regimen; LTR: long-term regimen; Xpert MTB/Rif Res: Refers to the Xpert MTB/Rif Resistance test, a molecular diagnostic test used to detect *Mycobacterium tuberculosis* and its resistance to Rifampicin; CAT-I: Refers to Category I of TB treatment, typically used for new, drug-sensitive TB patients with a standard 6-month regimen; CAT II: Refers to Category II of TB treatment, used for patients who have been previously treated for TB but experienced relapse, failure, or default, involving a more intensive treatment regimen.

Characteristic	Categories	Culture Conversion		HR	95% CI	P-value
		Yes	No			
All patients	138	107 (77.53)	31 (22.47)			
Age	< 36	49 (45.79)	14 (45.16)	REF		
	≥ 36	58 (54.21)	17 (54.84)	1.04	0.99-1.14	0.038
BMI	≥ 16 kg/m²	51 (47.66)	07 (22.58)	REF		
	< 16 kg/m²	56 (52.34)	24 (77.42)	1.07	0.94-1.29	0.043
Number of cigarette smoking per day	< 12	54 (50.47)	18 (58.06)	REF		
	≥ 12	53 (49.53)	13 (41.94)	1.02	0.97-1.33	0.031
Lung lesions	No	20 (18.69)	03 (9.68)	REF		
	Yes	87 (81.31)	28 (90.32)	1.19	0.93-1.65	0.008
Lung cavities	No	43 (40.19)	15 (48.39)	REF		
	Yes	64 (59.81)	16 (51.61)	0.81	0.71-1.11	0.029
Diabetes mellitus	No	77 (71.96)	25 (80.65)	REF		
	Yes	30 (28.04)	06 (19.35)	0.93	0.84-1.09	0.007
Sputum smear grades	≤ 1	49 (45.79)	17 (54.84)	REF		
	>1	58 (54.21)	14 (45.16)	1.03	1.96-1.38	0.041
Drug resistance tuberculosis	Xpert MTB/Rif Res	48 (44.86)	20 (64.52)	REF		
	MDR	59 (55.14)	11 (35.48)	1.31	1.01-1.89	0.002
Treatment history	No history of ATT	25 (23.36)	03 (9.68)	REF		
	CAT-I	70 (65.42)	18 (58.06)	0.31	0.19-0.59	0.048
	CAT-II	16 (14.95)	10 (32.26)			
Treatment Regimen Now	STR	56 (52.34)	20 (64.52)	REF		
	LTR	51 (47.66)	11 (35.48)	1.35	0.91-1.98	0.033
Antibiotics						
Moxifloxacin	Sensitive	95 (88.79)	29 (83.55)	REF		
	Resistant	12 (11.21)	02 (6.45)	1.77	0.95-3.29	0.040
Adverse effect						
Hepatotoxicity	No	72 (67.29)	23 (74.19)	REF		
	Yes	35 (32.71)	08 (25.81)	0.89	0.81-1.83	0.047
Anxiety	No	94 (87.85)	27 (87.10)	REF		
	Yes	13 (12.15)	04 (12.90)	1.33	1.21-2.51	0.038

**Table 8:** Multivariate analysis of factors influencing sputum conversion in non-smokers with drug-resistant tuberculosis patients P-value <0.05 is statically significant. STR: short-term regimen; LTR: long-term regimen; Xpert MTB/Rif Res: Refers to the Xpert MTB/Rif Resistance test, a molecular diagnostic test used to detect *Mycobacterium tuberculosis* and its resistance to Rifampicin; CAT-I: Refers to Category I of TB treatment, typically used for new, drug-sensitive TB patients with a standard 6-month regimen; CAT II: Refers to Category II of TB treatment, used for patients who have been previously treated for TB but experienced relapse, failure, or default, involving a more intensive treatment regimen.

Characteristic	Categories	Sputum Conversion		HR	95% CI	P-value
		Yes	No			
All patients	138	107 (77.53)	31 (22.47)			
Age	< 36	49 (45.79)	14 (45.16)	REF		
	≥ 36	58 (54.21)	17 (54.84)	1.02	0.99-1.12	0.038
Body mass index	≥ 16 kg/m²	51 (47.66)	07 (22.58)	REF		
	< 16 kg/m²	56 (52.34)	24 (77.42)	1.001	0.94-1.05	0.048
Living condition	Rural	94 (87.85)	17 (54.84)	REF		
	Urban	13 (12.15)	14 (45.16)	0.51	0.32-0.89	0.041
Number of cigarette smoking per day	< 12	54 (50.47)	18 (58.06)	REF		
	≥ 12	53 (49.53)	13 (41.94)	1.51	1.19-1.89	0.033
Lung lesions	No	20 (18.69)	03 (9.68)	REF		
	Yes	87 (81.31)	28 (90.32)	1.43	0.88-2.34	0.045
Lung cavities	No	43 (40.19)	15 (48.39)	REF		
	Yes	64 (59.81)	16 (51.61)	1.03	0.68-1.19	0.042
Diabetes mellitus	No	77 (71.96)	25 (80.65)	REF		
	Yes	30 (28.04)	06 (19.35)	0.68	0.45-0.91	0.039
Sputum smear grades	≤ 1	49 (45.79)	17 (54.84)	REF		
	>1	58 (54.21)	14 (45.16)	0.73	0.48-1.10	0.029
Drug resistance tuberculosis	Xpert MTB/Rif Res	48 (44.86)	20 (64.52)	REF		
	MDR	59 (55.14)	11 (35.48)	1.87	1.48-2.34	0.000
Treatment history	No history of ATT	25 (23.36)	03 (9.68)	REF		
	CAT-I	70 (65.42)	18 (58.06)	0.52	0.38-0.89	0.026
	CAT-II	16 (14.95)	10 (32.26)			
Treatment Regimen Now	STR	56 (52.34)	20 (64.52)	REF		
	LTR	51 (47.66)	11 (35.48)	1.47	1.00-2.16	0.046
Antibiotics						
Levofloxacin	Sensitive	83 (77.57)	26 (83.87)	REF		
	Resistant	24 (22.43)	05 (16.13)	1.74	1.09-2.76	0.018
Adverse Effects						
Nausea and vomiting	No	78 (72.90)	22 (70.97)	REF		
	Yes	29 (27.10)	09 (29.03)	0.62	0.40-0.96	0.034
Peripheral neuropathy	No	82 (76.64)	25 (80.65)	REF		
	Yes	25 (23.36)	06 (19.35)	0.50	0.31-0.80	0.004
Hepatotoxicity	No	72 (67.29)	23 (74.19)	REF		
	Yes	35 (32.71)	08 (25.81)	0.52	0.34-0.79	0.003
Arthralgia	No	81 (75.70)	23 (74.19)	REF		
	Yes	26 (24.30)	08 (25.81)	0.61	0.39-0.97	0.037

**Table 9:** Multivariate analysis of factors influencing culture conversion in non-smokers with drug-resistant tuberculosis patients P-value <0.05 is statically significant. STR: short-term regimen; LTR: long-term regimen; Xpert MTB/Rif Res: Refers to the Xpert MTB/Rif Resistance test, a molecular diagnostic test used to detect *Mycobacterium tuberculosis* and its resistance to Rifampicin; CAT-I: Refers to Category I of TB treatment, typically used for new, drug-sensitive TB patients with a standard 6-month regimen; CAT II: Refers to Category II of TB treatment, used for patients who have been previously treated for TB but experienced relapse, failure, or default, involving a more intensive treatment regimen.

Characteristic	Categories	Culture Conversion		HR	95% CI	P-value
		Yes	No			
All patients	138	107 (77.53)	31 (22.47)			
Age	< 36	49 (45.79)	14 (45.16)	REF		
	≥ 36	58 (54.21)	17 (54.84)	1.04	0.99-1.14	0.038
BMI	≥ 16 kg/m²	51 (47.66)	07 (22.58)	REF		
	< 16 kg/m²	56 (52.34)	24 (77.42)	1.07	0.94-1.29	0.043
Number of cigarette smoking per day	< 12	54 (50.47)	18 (58.06)	REF		
	≥ 12	53 (49.53)	13 (41.94)	1.02	0.97-1.33	0.031
Lung lesions	No	20 (18.69)	03 (9.68)	REF		
	Yes	87 (81.31)	28 (90.32)	1.19	0.93-1.65	0.008
Lung cavities	No	43 (40.19)	15 (48.39)	REF		
	Yes	64 (59.81)	16 (51.61)	0.81	0.71-1.11	0.029
Diabetes mellitus	No	77 (71.96)	25 (80.65)	REF		
	Yes	30 (28.04)	06 (19.35)	0.93	0.84-1.09	0.007
Sputum smear grades	≤ 1	49 (45.79)	17 (54.84)	REF		
	>1	58 (54.21)	14 (45.16)	1.03	1.96-1.38	0.041
Drug resistance tuberculosis	Xpert MTB/Rif Res	48 (44.86)	20 (64.52)	REF		
	MDR	59 (55.14)	11 (35.48)	1.31	1.01-1.89	0.002
Treatment history	No history of ATT	25 (23.36)	03 (9.68)	REF		
	CAT-I	70 (65.42)	18 (58.06)	0.31	0.19-0.59	0.048
	CAT-II	16 (14.95)	10 (32.26)			
Treatment Regimen Now	STR	56 (52.34)	20 (64.52)	REF		
	LTR	51 (47.66)	11 (35.48)	1.35	0.91-1.98	0.033
Antibiotic s						
Moxifloxacin	Sensitive	95 (88.79)	29 (83.55)	REF		
	Resistant	12 (11.21)	02 (6.45)	1.77	0.95-3.29	0.040
Adverse effect						
Hepatotoxicity	No	72 (67.29)	23 (74.19)	REF		
	Yes	35 (32.71)	08 (25.81)	0.89	0.81-1.83	0.047
Anxiety	No	94 (87.85)	27 (87.10)	REF		
	Yes	13 (12.15)	04 (12.90)	1.33	1.21-2.51	0.038

The time-dependent survival estimates for sputum and culture conversion reveal significant differences between smokers and non-smokers with DR-TB. Non-smokers show a rapid decline in survival proportions, with sputum conversion dropping to 0.000 by 90 days and culture conversion dropping to 0.000 by 70 days. In contrast, smokers exhibit a slower decline, with sputum conversion dropping to 0.137 by 120 days and culture conversion to 0.035 by 120 days (Table 10).

**Table 10:** Time-dependent survival estimates for smokers and non-smokers with drug-resistant tuberculosis

Characteristics	Time (Days)	Cumulative Survival Proportion		Cumulative Events	Remaining Cases
		Estimate	Std. Error		
Sputum Conversion					
Non-smoker	40	0.986	0.010	2	141
	44	0.930	0.021	10	133
	45	0.793	0.034	29	110
	50	0.742	0.037	36	101
	60	0.279	0.038	99	38
	65	0.215	0.036	107	27
	70	0.174	0.033	112	21
	80	0.078	0.024	123	9
	90	0.000	0.000	132	0
Smoker	80	0.993	0.007	1	134
	90	0.719	0.039	38	97
	100	0.510	0.044	65	66
	110	0.492	0.044	67	54
	120	0.137	0.032	106	15
Culture Conversion					
Non-smoker	25	0.783	0.034	31	112
	30	0.694	0.039	43	93
	35	0.545	0.043	63	73
	40	0.476	0.043	72	63
	45	0.393	0.042	83	52
	50	0.340	0.041	90	45
	60	0.062	0.021	126	8
	65	0.010	0.010	131	1
	70	0.000	0.000	132	0
Smoker	60	0.985	0.010	2	134
	80	0.956	0.018	6	129
	90	0.459	0.043	72	61
	100	0.209	0.040	96	20
	120	0.035	0.023	106	2

**Figure 1:**
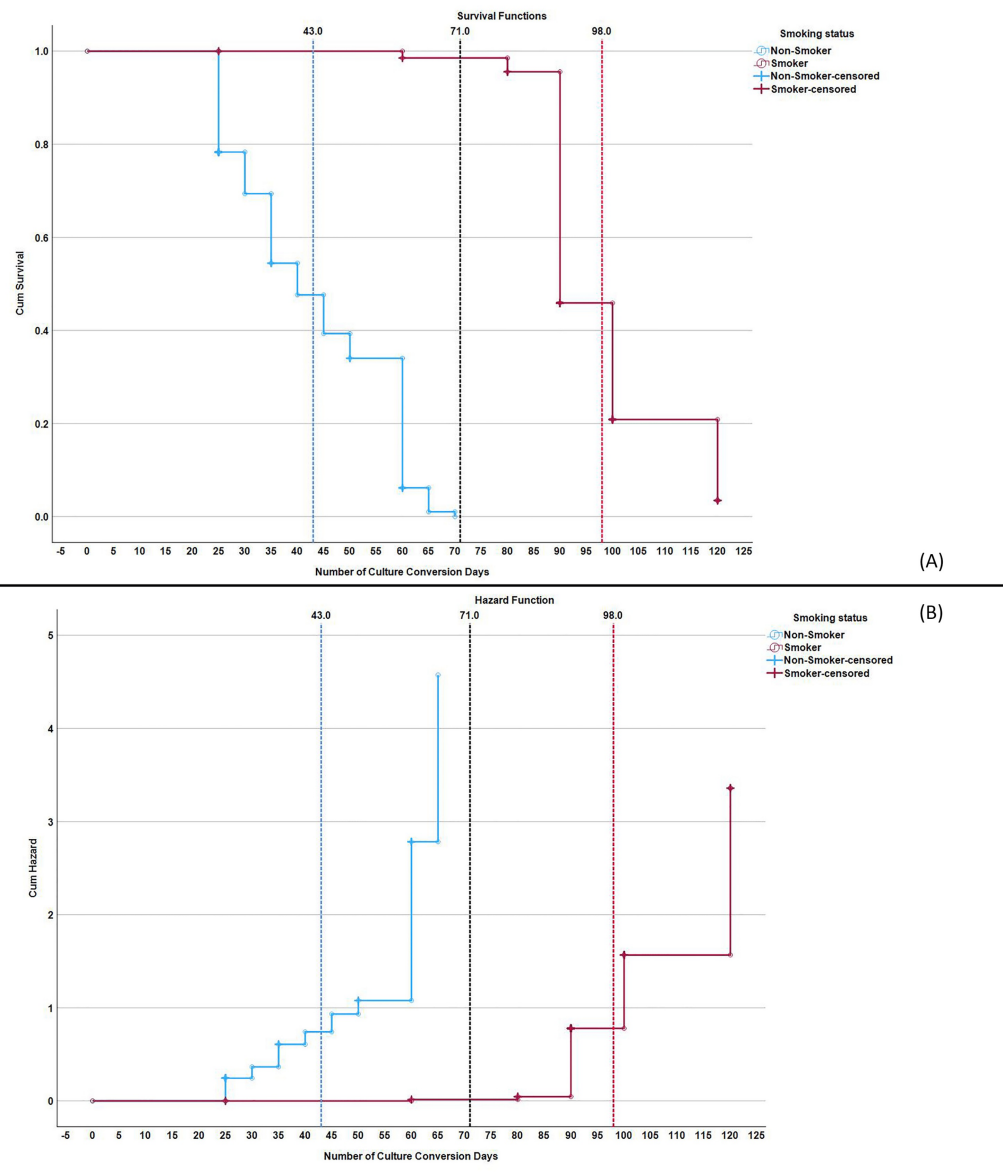
Kaplan-Meier curve of culture conversion times in smokers vs. non-smokers with drug-resistant tuberculosis Panel A: Survival Function – This panel shows the cumulative survival proportion over time (in days) for culture conversion, comparing smokers and non-smokers. The survival curve represents the probability of culture conversion at different time points. Panel B: Hazard Function – This panel shows the cumulative hazard function over time (in days) for culture conversion, comparing smokers and non-smokers. The hazard function represents the rate of failure (in this case, lack of culture conversion) over time.

**Figure 2:**
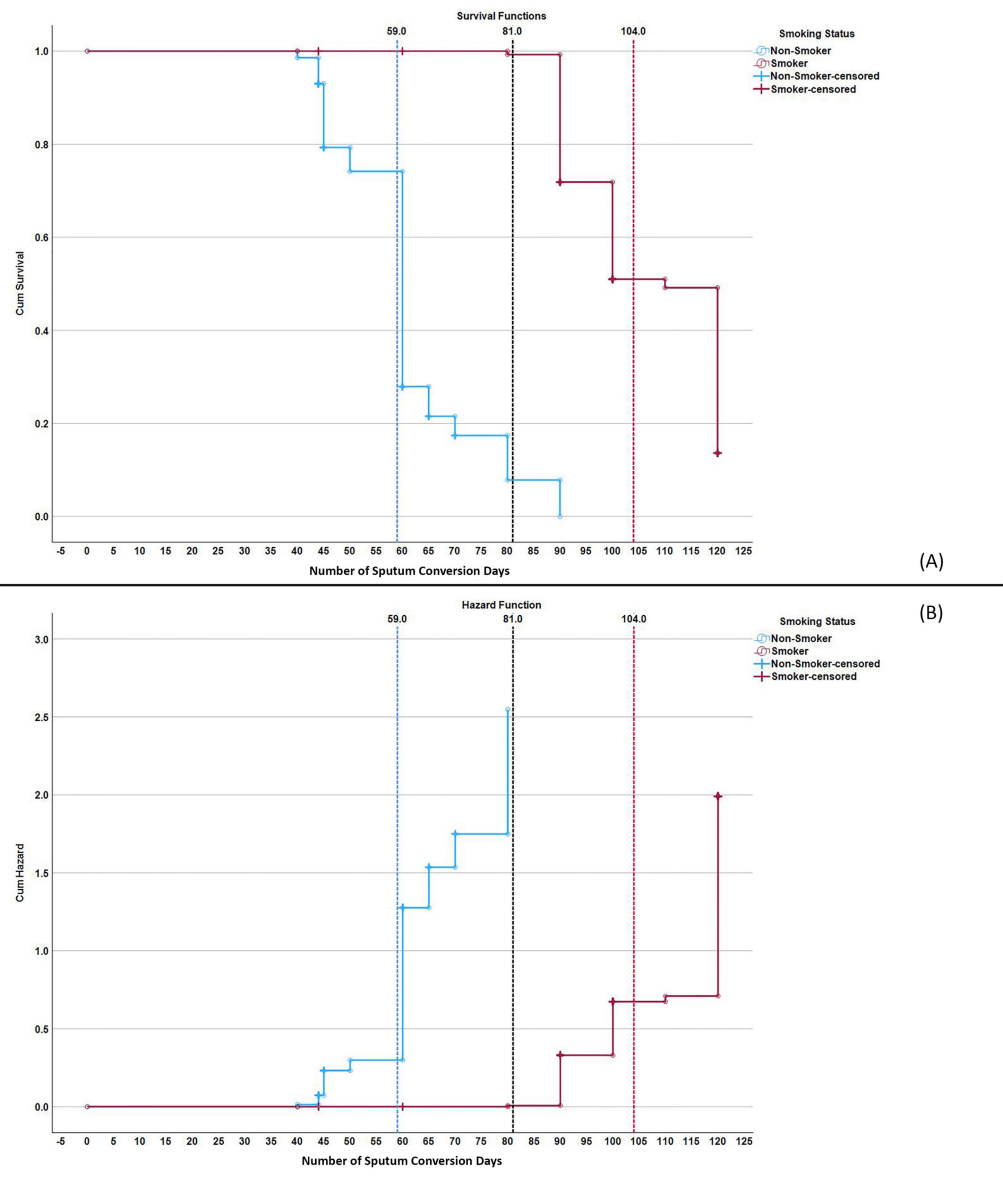
Kaplan-Meier curve of sputum conversion times in smokers vs. non-smokers with drug-resistant tuberculosis Panel A: Survival Function – This panel shows the cumulative survival proportion over time (in days) for sputum conversion, comparing smokers and non-smokers. The survival curve represents the probability of sputum conversion at different time points. Panel B: Hazard Function – This panel shows the cumulative hazard function over time (in days) for sputum conversion, comparing smokers and non-smokers. The hazard function represents the rate of failure (in this case, lack of sputum conversion) over time.

The graphs and mean sputum SCC times highlight a clear difference between smokers and non-smokers. Non-smokers tend to convert sputum faster, with a mean time of 59 days, compared to smokers who take 104 days on average. While for culture conversion non-smoker means time was 43 days and smoker 98 days. This significant delay in sputum SCC for smokers underscores the adverse impact of smoking on the treatment efficacy of DR-TB. The survival function for non-smokers shows a steep decline, indicating a higher and faster conversion rate, whereas smokers show a prolonged and gradual decline, signifying delayed conversion. The hazard function for smokers suggests a delayed increase in the risk of not converting, reflecting the prolonged treatment challenges faced by this group (Figures 1-2).

## Discussion

This study provides a comparative analysis of culture and sputum smear conversion timelines in smokers versus non-smokers with DR-TB. By focusing on the associated factors, including adverse effects, the study aims to explain how smoking influences treatment outcomes in DR-TB patients.

The baseline characteristics and clinical presentation of DR-TB patients in this study revealed distinct differences between smokers and non-smokers. Smokers were predominantly male, older, and had a lower mean body mass index (BMI) compared to their non-smoking patients. The demographic skew toward older males among smokers is consistent with patterns observed in broader epidemiological studies, which highlight higher smoking rates in these populations [10]. The lower BMI among smokers could be attributed to the well-documented association between smoking and malnutrition, where nicotine has been shown to suppress appetite and increase metabolic rates, leading to weight loss and nutritional deficiencies [11]. Clinically, smokers presented with more severe lung lesions, cavities, and higher sputum smear grades, indicating a more advanced disease state. This finding aligns with previous findings that smoking exacerbates pulmonary damage, thereby increasing the severity of TB presentation [12]. The more severe radiographic findings in smokers, such as extensive cavitary disease, suggest a direct link between smoking and the progression of TB pathology, possibly due to smoking-induced immune suppression and impaired pulmonary defense mechanisms [13].

The study also revealed notable differences in comorbidities and treatment histories between smokers and non-smokers. Smokers had a higher prevalence of diabetes and a history of previous TB treatment. [14] This finding is critical as it suggests a bidirectional relationship between smoking and comorbid conditions, where smoking exacerbates diabetes, and the presence of diabetes can complicate TB treatment. The higher incidence of previous TB treatment among smokers could indicate treatment failures or relapses, potentially driven by smoking-related immunosuppression and poor adherence to TB therapy [15]. Smokers also exhibited a higher prevalence of heart diseases compared to non-smokers. [16] This difference underscores the need for tailored management strategies that address the specific comorbid profiles of TB patients based on their smoking status. For smokers, cardiovascular health should be closely monitored and managed, therefore smoker comprehensive care plans should include diabetes management and strategies to prevent TB recurrence.

Treatment outcomes in this study were significantly poorer for smokers, with lower success rates, higher mortality, and delayed sputum and culture conversion times compared to non-smokers. These findings are consistent with a substantial body of literature documenting the adverse effects of smoking on TB treatment efficacy and patient survival [7]. The delayed sputum and culture conversion times among smokers suggest that smoking may impair the bactericidal activity of TB drugs, potentially through mechanisms involving altered drug metabolism and reduced immune responses. Higher rates of treatment failure and adverse effects among smokers highlight the need for specialized support systems. Smoking cessation programs should be integrated into TB treatment regimens to improve adherence and outcomes. Additionally, adherence strategies tailored to smokers, such as counseling and pharmacotherapy for nicotine dependence, could significantly enhance treatment success rates.

The study identified higher resistance to key antibiotics among smokers, necessitating more complex treatment regimens and potentially impacting treatment outcomes. This finding is particularly concerning as it indicates that smoking may be associated with an increased risk of developing drug-resistant TB strains [17]. The underlying mechanisms could include impaired drug absorption, altered pharmacokinetics, and compromised immune responses in smokers, all of which contribute to the development and persistence of resistant TB strains. The adverse effects profile also differed significantly between smokers and non-smokers. Smokers experienced more frequent and severe side effects, which could be attributed to variations in drug metabolism and the compounding toxic effects of smoking on the liver and kidneys [18]. These differences underscore the importance of monitoring and managing adverse effects closely in smokers to prevent treatment discontinuation and ensure successful outcomes.

The multivariate analysis in this study identified several factors influencing sputum and culture conversion rates in both smokers and non-smokers. Key factors included the severity of lung lesions, the presence of comorbidities such as diabetes, and previous treatment history. [19] The survival and hazard analysis further highlighted prolonged conversion times in smokers, suggesting a delayed response to treatment compared to non-smokers. This delay could be due to the more advanced disease state and higher prevalence of drug resistance among smokers. The findings underscore the need for tailored treatment strategies that address these differences effectively, such as personalized medication regimens and intensified monitoring for smokers.

The study has several limitations that warrant consideration. Firstly, the research was conducted at a single medical center, Mardan Medical Complex, which restricts the generalizability of the findings to other settings with diverse patient demographics, healthcare systems, and resource availability. Multicenter studies would yield more robust and widely applicable insights. Secondly, all smoker patients in this study were male, introducing a significant gender bias and limiting the applicability of the findings to female smokers. Gender differences in smoking behavior, disease progression, and treatment response are well-documented, and the absence of female participants may overlook important gender-specific factors influencing DR-TB outcomes. Thirdly, the exclusion of patients with incomplete medical records, co-infections (e.g., HIV, COVID-19), major comorbidities (e.g., cancer, autoimmune diseases), and recent surgeries could skew the study results. These exclusions may omit individuals more likely to experience severe outcomes, potentially underestimating the true impact of smoking on DR-TB treatment. Fourthly, the study does not monitor changes in smoking behavior over time. Smoking cessation during treatment could alter outcomes, and without longitudinal data, it is challenging to assess the dynamic impact of smoking cessation or relapse on treatment efficacy and adverse effects. Finally, the study does not analyze specific biomarkers that could provide insights into the physiological differences between smokers and non-smokers, such as markers of inflammation, immune function, and metabolic changes. Incorporating biomarker analysis could enhance the understanding of the biological mechanisms underlying the observed differences in treatment outcomes.

## Conclusions

This study demonstrates that smoking impairs immune function and affects the pharmacokinetics of anti-tuberculosis drugs, leading to extended and complex treatment courses for patients with DR-TB. The delayed culture and sputum smear conversion observed in smokers suggest ongoing bacterial activity and elevated transmission risks, posing significant challenges to TB control efforts. Smokers are more likely to be older, have a lower BMI, and exhibit severe lung lesions and cavities. Additionally, they often present with multiple comorbidities and experience more severe adverse effects from treatment, all of which contribute to prolonged conversion timelines. Addressing these issues through personalized treatment regimens and comprehensive care plans is essential for optimizing outcomes in this patient population.
